# Phenotypic Environmental Sensitivity and Mental Health During Pregnancy and Post Partum: Protocol for the Experiences of Pregnancy Longitudinal Cohort Study

**DOI:** 10.2196/49243

**Published:** 2023-12-06

**Authors:** Charlie Rioux, Delaney C Fulp, Parker N Haley, Jenna L LaBelle, Mary E Aasted, Kasie K Lambert, Madison T Donohue, Nkatheko T Mafu

**Affiliations:** 1 Department of Psychology University of Oklahoma Norman, OK United States; 2 Jeannine Rainbolt College of Education University of Oklahoma Norman, OK United States

**Keywords:** anxiety, biological sensitivity to context, depression, diathesis-stress, highly sensitive person, moderation, resilience, sensory processing reactivity, substance use, vantage sensitivity

## Abstract

**Background:**

Mental health problems during pregnancy and post partum are common and associated with negative short- and long-term impacts on pregnant individuals, obstetric outcomes, and child socioemotional development. Socio-environmental factors are important predictors of perinatal mental health, but the effects of the environment on mental health are heterogeneous. The differential susceptibility theory and the environmental sensitivity framework suggest that individuals differ in their degree of sensitivity to positive and negative environments, which can be captured by individual phenotypes such as temperament and personality. While there is strong evidence for these models in childhood, few studies examined them in adults, and they were not examined in pregnancy.

**Objective:**

The primary objective of the Experiences of Pregnancy study is to explore whether childhood and current environments are associated with mental health and well-being in pregnancy and whether these effects depend on individual sensitivity phenotypes (personality). This study also aims to gather important psychosocial and health data for potential secondary data analyses and integrative data analyses.

**Methods:**

We will conduct a longitudinal cohort study. The study was not registered elsewhere, other than this protocol. Participants will be recruited through social media advertisements linking to the study website, followed by an eligibility call on Zoom (Zoom Video Communications). Participants must be aged 18 years or older, currently residing in the United States as citizens or permanent residents, and currently planning to continue the pregnancy. A minimum of 512 participants will be recruited based on power analyses for the main objectives. Since the data will also be a resource for secondary analyses, up to 1000 participants will be recruited based on the available budget. Participants will be in their first trimester of pregnancy, and they will be followed at each trimester and once post partum. Data will be obtained through self-reported questionnaires assessing demographic factors; pregnancy-related factors; delivery, labor, and birth outcomes; early infant feeding; individual personality factors; childhood and current environments; mental health and well-being; attachment; and infant temperament. A series of measures were taken to safeguard the study from web robots and fraudulent participants, as well as to reduce legal and social risks for participants following *Dobbs v. Jackson*.

**Results:**

The study received ethics approval in April 2023 from the University of Oklahoma-Norman Campus Institutional Review Board. Recruitment occurred from May to August 2023, with 3 follow-ups occurring over 10 months.

**Conclusions:**

The Experiences of Pregnancy study will extend theories of environmental sensitivity, mainly applied in children to the perinatal period. This will help better understand individual sensitivity factors associated with risk, resilience, plasticity, and receptivity to negative and positive environmental influences during pregnancy for pregnant individuals.

**International Registered Report Identifier (IRRID):**

PRR1-10.2196/49243

## Introduction

### Background

Pregnancy is a period of increased risk for mental health problems, which are one of the leading preventable causes of morbidity and mortality during the perinatal period in high-income nations [1-3]. Among people who are pregnant, approximately 20% to 30% experience elevated symptoms of anxiety or depression [4-6]. In turn, these symptoms are associated with short- and long-term parental and child health and well-being. Elevated and sustained prenatal psychological distress can often lead to long-term disturbing emotional experiences such as anger, anxiety, and depression in the postpartum period and beyond [7-9]. Prenatal stress and distress may impair bonding with the fetus and child [10,11] and may lead to strained familial and social relationships. In infants, exposure to prenatal stress and mental health problems has been found to be associated with a more difficult temperament and impaired cognitive and motor development [12]. Impacts on children may be long-term, with prenatal stress and distress being associated with disorganized attachment, poorer socioemotional development, learning deficits, and mental health problems across the internalizing and externalizing spectrums in children and adolescents, including attention-deficit/hyperactivity disorder, anxiety, depression, and behavioral problems [12-15]. Finally, prenatal stress and distress are also associated with obstetric and birth outcomes, with research showing links with early gestational age, low weight at birth, preeclampsia, hypertension, and reduced lactation [8,12,16-19].

Considering these broad negative impacts, understanding the risk and protective factors for mental health problems during pregnancy, as well as factors associated with well-being, is essential to promoting parental and child health. Environmental factors, both in early life and during pregnancy, have been found to be associated with perinatal mental health. For example, a meta-analysis found that adverse childhood experiences are a risk factor for prenatal and postpartum depression and anxiety, with the number of adverse childhood experiences being positively associated with symptom severity [20]. Sociodemographic factors are also important, with a meta-analysis showing that lower educational level and poor economic status are risk factors for perinatal depression [21]. Finally, research on current social environment factors shows that low social support from family members, friends, or community members [22], as well as poor romantic relationship satisfaction [23,24], are risk factors for perinatal depression, anxiety, and self-harm. The effects of the environment on mental health are, however, heterogeneous.

A proper understanding of the associations between environmental factors and mental health requires studying for whom the environment is influential. In turn, this can inform better targeted prevention efforts when psychosocial resources are limited. A frequently used theoretical model in psychiatry is the diathesis-stress model of vulnerability and resilience [25,26]. According to this model, some pregnant individuals would be vulnerable to adverse environments, leading to higher levels of psychological distress, while others would be resilient to such environments, leading to low levels of psychological distress despite negative environmental conditions. An alternative model, the differential susceptibility model [27,28], suggests that pregnant individuals who are sensitive to their environment would have higher levels of psychological distress than their peers in adverse environments but also lower levels of psychological distress and higher levels of well-being than their peers in positive environments. Thus, rather than being only vulnerable to adverse environments, individuals could be sensitive to both positive and negative environments, “for better or for worse” [29]. Individuals could also, however, be sensitive only to positive environmental influences, which was proposed in the vantage sensitivity model [30].

**Figure 1:**
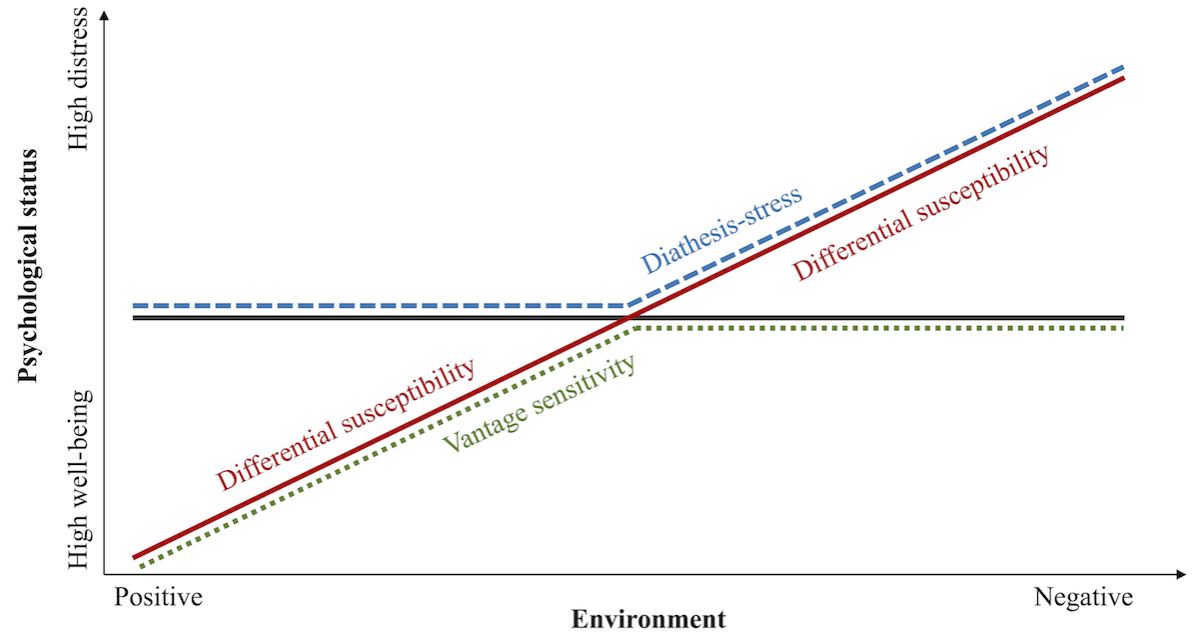
Integrated model of environmental sensitivity. The diathesis-stress model captures individual sensitivity to negative environments only. The vantage sensitivity model captures individual sensitivity to positive environments only. The differential susceptibility captures sensitivity to both negative and positive environments within the same individuals (adapted from Belsky et al [32], which is published under Creative Commons Attribution 4.0 International License [41]).

The environmental sensitivity framework [31] (Figure 1 [32]) comprehensively integrates these theoretical models of psychosocial person-environment interactions. According to this model, individuals differ in their degree of sensitivity to the environment. Based on an individual sensitivity factor, individuals may differ in their sensitivity to adverse or negative environments only (diathesis-stress), to enriched or positive environments only (vantage sensitivity), or to both (differential susceptibility) [31]. Furthermore, some evidence suggests that this sensitivity may be domain specific rather than domain general, meaning different individual characteristics may capture sensitivity to different environments [33]. It is thus important to further elucidate for whom, when, and under which environmental conditions diathesis-stress, vantage sensitivity, and differential susceptibility apply. This is important because individuals’ differences in their receptivity to environmental inputs can influence their response to treatment interventions [34]. A better understanding of environmental sensitivity can thus help determine the best intervention and treatment on an individual basis, as well as inform targeted prevention efforts and psychoeducation [34,35].

The differential susceptibility and environmental sensitivity models were proposed by developmental psychologists and have been thoroughly studied in children, with several reviews showing that individual-level factors originally thought to be vulnerability factors to adverse environments supported differential susceptibility, reflecting sensitivity to certain positive and negative environments [27,28,36]. We still know very little, however, about how these models apply to adults. Theory suggests that some adults are sensitive to positive and negative environmental conditions in the prediction of mental health, but very few empirical studies have examined the patterns of interaction of the environmental sensitivity framework based on phenotype in adults [37-39]. Thus, research is needed to elucidate environmental sensitivity factors in adulthood, including during pregnancy, a key period of heightened risk for psychological distress [1,5,40].

### Objectives

The primary objective of the Experiences of Pregnancy study is to explore whether childhood and current environments are associated with mental health and well-being in pregnancy and whether these effects depend on individual sensitivity phenotypes (personality) through a longitudinal cohort study design. Considering there are few empirical studies on this topic in adulthood in general and no data to our knowledge in pregnancy, the study aims to document a variety of individual, environmental, and mental health factors to clarify models of sensitivity in adults and pregnant people. Following epidemiological cohort principles, this study also aims to gather important psychosocial and health data for potential secondary data analyses [42] and integrative data analyses [43].

## Methods

### Participants and Recruitment

**Figure 2:**
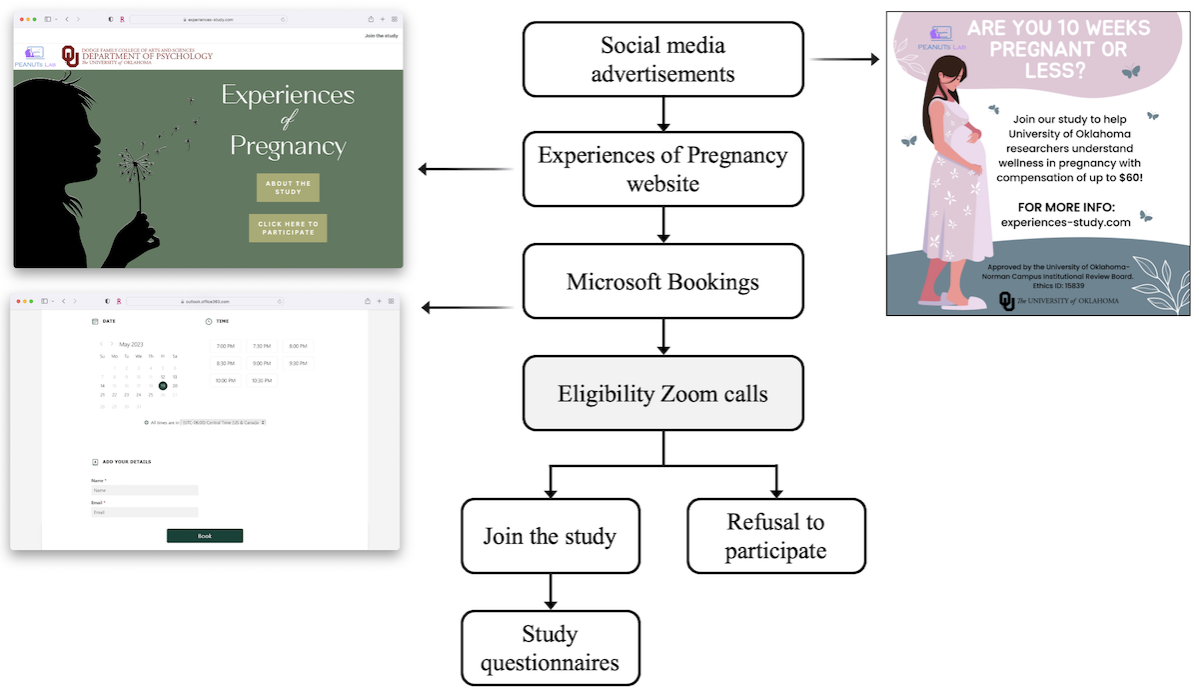
Flowchart of the study procedure for participant enrollment for the Experiences of Pregnancy longitudinal cohort study, United States, planned for 2023-2024.

Figure 2 shows the recruitment process to occur from May to August 2023. Participants will be recruited on the web through advertisements (Figure 2) on Facebook, Instagram, Twitter (subsequently rebranded X), and Reddit, as well as posts on the Reddit forums “r/Maternity” and “r/lgbtstudies.” Participants will be directed to the study website [44], where they will find general information about the study, eligibility criteria, and a link to join the study. Through this link, interested participants will be directed to a REDCap (Research Electronic Data Capture; Vanderbilt University) [45,46] survey protected by a reCAPTCHA Inc (Google) verification and redirecting to a Microsoft Bookings (Microsoft Corporation) page to schedule their call, with availabilities open for the next 14 days. Zoom (Zoom Video Communications) calls with undergraduate and graduate research assistants (RAs) will confirm their eligibility and go over the study process. Eligible and interested participants will receive their personal survey link through email after their call.

Participants are eligible to enroll if they are aged 18 years or older, currently live in the United States, are citizens or permanent residents of the United States, and can read and write in English. Pregnant people of all gender identities are eligible to enroll in the study [47]. Furthermore, participants have to be currently planning to continue the pregnancy (see the *Ethical Considerations* section) and be in their first pregnancy trimester to join the study. Eligibility is to be 10 weeks pregnant or less when they signal their interest in the study (make their appointment through Microsoft Bookings) to allow time for their Zoom call confirming eligibility and for filling out the first survey. They remain eligible if they are 12 weeks pregnant or less by the time of their Zoom call. Overall, this allows for 2 weeks to schedule the Zoom call and gives them at least 1 week to fill out the first survey.

### Follow-Ups and Study Design

**Figure 3:**
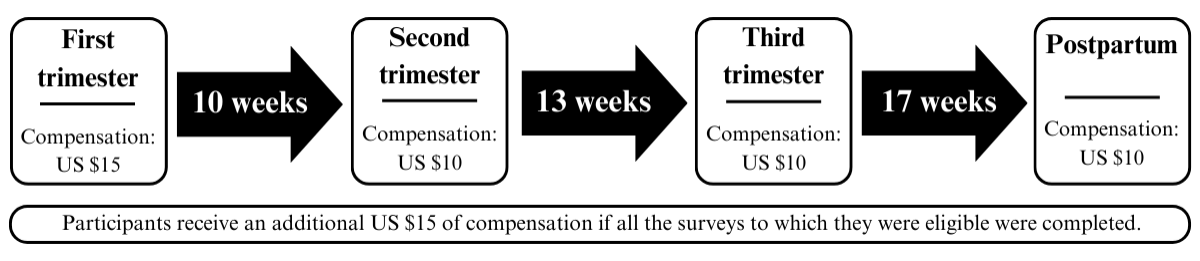
The timeline of completion of the study questionnaires for the Experiences of Pregnancy longitudinal cohort study, United States, planned for 2023-2024.

Participants will fill out 4 questionnaires: 1 questionnaire per trimester of pregnancy (3 first questionnaires) and 1 after birth (Figure 3). This follow-up schedule will allow collection of data during each key developmental period (trimester) of pregnancy. For this study, this follow-up schedule was also based on research showing individual differences in how mental health changes across each trimester [48-51] and highlighting the importance of repeated measurements at each pregnancy trimester in assessing prenatal mental health.

At each follow-up survey, participants will first be asked if they are still pregnant. In the event that a participant experiences pregnancy loss or premature birth, they will remain eligible to fill out the current survey as their last assessment (ie, 1 survey after loss or after birth). Depending on group sample sizes, this may allow for the examination of mental health and resilience factors after a pregnancy loss. At the end of each survey, the “submit” button redirects participants to resources on the study website, with resource lists for pregnancy, pregnancy loss, and post partum that include crisis lines, mental health services, information, and networks.

Based on an estimate of 4 to 7 items per minute for participants who do not regularly participate in survey studies [52], the first survey is expected to take between 50 and 70 minutes. The following 3 questionnaires are expected to take between 40 and 60 minutes; participants will be informed of these time estimates during their Zoom call. Based on the upper range of US minimum wages, participants will be compensated for their time, receiving US $15 of compensation for the first questionnaire (longer questionnaire) and US $10 for each subsequent questionnaire. As an incentive to complete the study and to reduce attrition, participants who complete all 4 questionnaires (or all questionnaires for which they are eligible in the event of a pregnancy loss or premature birth) will receive an additional US $15. Compensation will be in the form of Amazon or Giftogram e-gift cards sent by email within 2 weeks of each questionnaire’s completion. To receive compensation, participants must submit the survey but are free not to answer questions they are uncomfortable with or do not wish to answer.

### Study Measures

All study data will be obtained through self-reported questionnaires. Table 1 summarizes study measures across assessments. All personality measures will be collected at survey 1 only as potential moderators. Childhood environments will be retrospectively self-reported once as potential predictors. Adverse and benevolent childhood experiences will be assessed at survey 1, but parental bonding (a retrospective measure of the parenting styles of participants’ parents in their childhood) will be assessed at survey 2 to reduce the burden of the longer first questionnaire. Current environments will be measured repeatedly at each assessment and include recent stressful experiences, everyday discrimination, social support, couple satisfaction, romantic intimate bonding, and social and digital media use. Mental health and well-being measures will be measured repeatedly at each assessment and include depressive symptoms, anxiety symptoms, anger, well-being, life satisfaction, sleep, disturbed dreaming, substance use (alcohol, tobacco, cannabis, and other drugs), and mental health service use. The initial (survey 1) substance use questionnaires will also assess substance use in the year before pregnancy as well as the age of onset for each substance. Attachment (prenatal or postnatal) will also be measured at all assessments. Finally, infant temperament will be measured after birth. Both postnatal attachment and infant temperament will be measured only for singleton pregnancies, as these questionnaires are specific to 1 baby, and the surveys would be much longer if asked for multiple babies.

**Table 1:** Study measures at each assessment for the Experiences of Pregnancy longitudinal cohort study, United States, planned for 2023-2024.

Study measures					Survey 1	Survey 2	Survey 3	Survey 4
					First trimester	Second trimester	Third trimester	Post partum
**Demographic, pregnancy, and delivery information**								
		Demographic information			All^a^			
		Relationship status			All	All	All	All
		Daily activities and responsibilities			All	All	All	All
		Pregnancy status				All	All	All
		Previous pregnancies and due date			All			
		Pregnancy intendedness [53]			All			
		Health conditions before pregnancy			All			
		Health conditions during pregnancy			All	P^b^ and LD^c^	P and LD	LD
		Height			All			
		Weight			All	P and LD	P and LD	
		Prenatal care			All	P and LD	P and LD	
		Delivery and birth outcomes				LD	LD	LD
		Early breastfeeding and formula feeding				LD	LD	LD
		Labor and Delivery Index [54]				LD	LD	LD
**Individual and personality factors**								
		10-item Big Five Inventory [55]			All			
		Highly Sensitive Person 12-item Scale [56]			All			
		Substance Use Risk Profile Scale [57]			All			
**Environmental factors**								
				Expanded Adverse Childhood Experiences Scale [58]	All			
				Benevolent Childhood Experiences Scale [59]	All			
				Parental Bonding Instrument [60]		All		
				Recent Stressful Experiences Checklist [61]	All	All	All	All
				Everyday Discrimination Scale [62]	All	All	All	All
				Multidimensional Scale of Perceived Social Support [63]	All	All	All	All
				Couple Satisfaction Index [64]	All	All	All	All
				Intimate Bond Measure [65]	All	All	All	All
				Social and digital media use	All	All	All	All
**Mental health and well-being measures**								
				PROMIS^d^ Anger [66]	All	All	All	All
				Generalized Anxiety Disorder 7-item scale (GAD-7) [67]	All	All	All	All
				Depression: Patient Health Questionnaire (PHQ-9) [68]	All	All	All	All
				Substance use	All	All	All	All
				Psychological Well-Being Scale [69]	All	All	All	All
				PROMIS General Life Satisfaction [70]	All	All	All	All
				Sleep duration	All	All	All	All
				PROMIS Sleep Disturbance [71]	All	All	All	All
				PROMIS Sleep-Related Impairment [71]	All	All	All	All
				Disturbing Dreams and Nightmare Severity Index [72]	All	All	All	All
				Mental Health Service Use Questionnaire [73]	All	All	All	All
**Attachment**								
		Maternal Antenatal Attachment Scale [74]			All	P	P	
		Maternal Postnatal Attachment Scale [75]				LD	LD	LD
**Infant temperament**								
			Early Infancy Temperament Questionnaire [76]^e^			LD	LD	LD
**Other**								
	Intention to participate in follow-up surveys^f^				All	P		

^a^All: study measure is included in regular follow-ups and shown to all participants regardless of pregnancy status.

^b^P: pregnant; this indicates study measure is included for participants who are still pregnant at the time of that questionnaire.

^c^LD: live delivery; this indicates study measure is included for participants who experienced a (term or preterm) live delivery at the time of that questionnaire. Participants who experience a pregnancy loss or neonatal death are only shown instruments labeled “All” and are not shown instruments related to pregnancy, labor, delivery, or the child.

^d^PROMIS: Patient-Reported Outcomes Measurement Information System.

^e^Only the subscales “Rhythmicity,” “Intensity,” and “Threshold” are included.

^f^See the *Missing Data Mitigation* section for details.

### Sample Size Considerations

We aim to recruit between 512 and 1000 participants. This study’s main objective is to explore person-environment interactions in the prediction of perinatal mental health and well-being. We examined power for the simplest model to meet these objectives (1 interaction effect in a multiple regression model). Power analyses were run using the *InteractionPoweR* Shiny app [77], with moderate main effect sizes and small interaction effect sizes based on our previous differential susceptibility research [78-80]. Measurement reliability was integrated into the power analyses, with good (environmental and personality variables) and excellent (mental health) reliabilities [23,56,80,81]. This analysis indicated that the minimum sample size to detect the interaction effects with 80% power is 341. Simulation studies suggest that for tests of differential susceptibility, 50% more cases are required to achieve 80% power compared with power for the interaction effect [82], yielding a minimum sample size of 512. This study is also designed to serve as a secondary data analyses related to mental health and well-being during pregnancy and post partum. To maximize possibilities for longitudinal modeling, we will be recruiting up to 1000 participants, with the maximum being based on budgetary considerations.

### Web-Based Data Integrity

Measures were used to safeguard the study against web robots (bots). To prevent enrollment from bots and fraudulent participants, participants will be individually screened through Zoom calls. Individually screening participants has been deemed one of the most reliable methods to protect web-based data collections [83,84]. Considering recent advances in artificial intelligence (AI) language, calls were deemed more secure than emails for participant screening. Zoom calls were easier to implement administratively than phone calls, as there were already organizational accounts set up for Zoom, but phone accounts had to be purchased. Finally, appointments were expected to be more efficient than trying to reach participants through phone calls after they provided their information without a set time.

To safeguard the Zoom call bookings from being targeted by bots, which could prevent genuine participants from enrolling due to appointments being unavailable, a total of 4 measures recommended to deter bots from studies were used. First, because bots are particularly interested in studies with financial incentives [83], monetary compensation was included in the images of the social media advertisements but will not be mentioned in any of the text accompanying the advertisements. Second, a total of 2 links will have to be accessed to get to the call bookings (the social media advertisement link and the link to join the study on the website). Direct links to Microsoft Bookings will not be posted on social media [83,85]. Third, a reCAPTCHA verification will protect the REDCap survey leading to the bookings page [83,85]. Finally, Microsoft Bookings settings will include 1-time passwords, where a numeric code will be sent to each participant’s email address to complete the booking.

RAs conducting Zoom calls will complete 2 trainings. The first training (in person) will go over the study protocol, the steps for confirming calls with participants through email, conducting the call, and sending surveys, including how to use Microsoft Bookings, the study email account, and REDCap. Each RA will have a printed copy of a manual covering all these steps, along with access to a digital version of the manual. RAs will also practice all steps, including the calls, during the first training. The second training will be on Zoom, where RAs will practice calls further in a more similar setting to calls with participants. The principal investigator (CR) will have weekly scheduled meetings with RAs to discuss progress, questions, and any perceived issues during the calls, including potential fraud. RAs will also be able to share any perceived issues with a call in a rapid manner through the laboratory’s communication platform, Slack (Slack Technologies). This will allow the identification of any unforeseen issues during recruitment and the adaptation of call protocols if needed.

### Missing Data Mitigation

To reduce attrition rates, an incentive of US $15 is offered for completing all 4 surveys (or all surveys a participant is eligible to in the event of a pregnancy loss or premature birth; see the *Follow-Ups and Study Design* section). Because participants are actively recruited and join the study through a Zoom call instead of only filling out the survey on the web, we expect more participant investment in the study than traditional web-based questionnaires.

In analyses with missing data rates up to 10%, deletion techniques may be used as they are not associated with increased bias [86]. In analyses above this missing data rate (which can be expected for longitudinal analyses), recommended missing data treatments, including full information maximum likelihood (FIML) and multiple imputation, will be used [87]. These missing data treatments are most effective at preventing biased results when predictors of missing data (ie, predictors of the missing at random [MAR] mechanism) are included as auxiliary variables [87]. At the first 2 assessments, we provided a study timeline of the next surveys and their associated compensation and included 1 question asking participants “Currently, how likely do you think you are to complete the follow-up surveys?” with answers ranging from “very unlikely” (score 1) to “very likely” (score 4), presented in Table 1. This question was included to serve as a potential predictor of the MAR mechanism (ie, potential auxiliary variable) in addition to the main survey questions.

### Data Management

All data will be collected and managed using REDCap tools hosted at the University of Oklahoma Health Sciences Center [45,46]. REDCap is a secure, web-based software platform designed to support data capture for research studies, providing (1) an intuitive interface for validated data capture, (2) audit trails for tracking data manipulation and export procedures, (3) automated export procedures for seamless data downloads to common statistical packages, and (4) procedures for data integration and interoperability with external sources. Metadata will be submitted to the Maelstrom Catalogue [88] to support harmonization initiatives among epidemiological cohorts. The research team welcomes collaboration on the study; access to the data dictionary and data request form will be available upon reasonable request made to the corresponding author.

### Data Analyses

Analyses for the primary aim will follow recommendations for testing person-environment interactions based on the environmental sensitivity framework and differential susceptibility. Linear moderated regressions will be conducted. To test for diathesis-stress, vantage sensitivity, and differential susceptibility, the nature of significant interactions will be further examined using regions of significance analyses, the proportion of interaction (PoI) index, and the proportion affected (PA) index [89]. The best-practice recommendations are to use a combination of these indices to confirm support for one of the environmental sensitivity models [82,89].

Regions of significance analyses use the Johnson-Neyman technique to identify at what levels of the environmental variable individuals differ in their mental health based on their phenotype [89]. The PoI index will quantify the “proportion of the total area of the interaction” that is attributable to the positive environment. Prototypical models would have a PoI of 0.5 for differential susceptibility, 0.0 for diathesis-stress, and 1.0 for vantage sensitivity. Proposed criteria are that a PoI of 0.2-0.8 supports differential susceptibility [82], with a PoI <0.2 supporting diathesis-stress and a PoI >0.8 supporting vantage sensitivity. The PA index will quantify the “proportion of participants” who benefit from the positive environment. Similar to the PoI index, prototypical models would have a PA of 0.5 for differential susceptibility, 0.0 for diathesis-stress, and 1.0 for vantage sensitivity. Proposed criteria are that a PA value <0.16 is considered as indicative of diathesis–stress [89], making a PA value >0.84 indicative of vantage sensitivity.

### Ethical Considerations

The study received ethics approval from the University of Oklahoma-Norman Campus Institutional Review Board (IRB #15839). All participants will sign an electronic informed consent form once during the study through the REDCap eConsent framework [90] before completing the first questionnaire.

Special considerations were taken to reduce the legal and social risks and protect participants from their data being used in legal proceedings related to abortion. First, a National Institutes of Health Certificate of Confidentiality was obtained. Second, inclusion criteria explicitly include the intention to keep the pregnancy, so that pregnancy losses recorded in the study would not be assumed to be from intentional abortion. Third, while our pregnancy-information questionnaire would have typically included the following questions—(1) How many total times have you been pregnant? (2) Have you ever had a miscarriage? and (3) How many children do you currently have at home?—in order to avoid combined answers to these questions being used to deduce the possibility of a past abortion, questions 2 and 3 were removed, and question 1 was changed to “Is this your first pregnancy?” Fourth, while we initially planned an open-ended question following pregnancy losses where participants could tell us about their experience, we opted to remove that open-text field to avoid inadvertently recording liable information.

## Results

The study received ethics approval in April 2023. Recruitment occurred from May to August 2023. After the first survey, participant follow-ups will occur over 10 months.

## Discussion

The Experiences of Pregnancy study will assess individual personality factors, social environmental factors, and psychological distress and well-being factors throughout pregnancy and post partum. The Experiences of Pregnancy study will allow extending theories of environmental sensitivity mainly applied in children to the perinatal period. This will help better understand individual characteristics associated with risk, resilience, plasticity, and receptivity to negative and positive environmental influences during pregnancy. In turn, this could help inform intervention and prevention efforts tailored to peoples’ individual sensitivity to their environment [34,35]. While measures on individual phenotypes, social environments, and mental health outcomes were prioritized, the principal investigator’s (CR) expertise in longitudinal study designs, population studies, and advanced quantitative analyses allowed the integration of a broader set of measures that will allow secondary data analyses and could contribute to data harmonization efforts addressing related developmental and epidemiological questions.

Recruitment for Experiences of Pregnancy is occurring nationwide, is completely web based, and will be carefully tracked. The infiltration of web-based data by bots and fraudulent participants has been a major concern in recent years [91,92]. In this study, we implemented many steps to ensure data integrity and protect the study from bots and fraudulent participants while ensuring feasibility, including not linking surveys directly on social media, reCAPTCHA verification, email verification, and a Zoom call. The study has the potential to contribute to the design of future studies looking to conduct reliable and safe web-based data collection. This could help future epidemiological and medical research since web-based data collection can have advantages in collecting data from minority populations and from hard-to-reach populations [93-96]. This is not limited to self-report surveys such as ours since many aspects of data collection, including, for example, some biological assays [97-100], neurobehavioral and cognitive assessments [101-103], stress tests [104,105], and parent-child observations [106], have been successfully adapted to be conducted remotely. Finally, our data on social media advertisement costs and success will also be documented and could help inform recruitment budgets for future web-based studies on pregnant populations.

The main weakness of the Experiences of Pregnancy study at the design stage is that all data will be self-reported. However, initial insights into environmental sensitivity and differential susceptibility in pregnancy stemming from this study could inform future investments in research integrating other measurement methods, such as examining genotypic and endophenotypic sensitivity factors [28,107]; recruiting participants’ social network (eg, parents, siblings, friends, and romantic partners) to measure their social environment through multiple informants [108]; integrating objective stress measures; or accessing medical record information for participants’ health, mental health, and birth outcomes. The generalizability of the results stemming from this study will also be limited to English-speaking US residents and citizens.

Overall, the Experiences of Pregnancy study will help improve our understanding of risk and protection factors for perinatal distress, as well as how different factors interact with each other to predict mental health and well-being. In turn, this can help inform efforts aiming to reduce the high prevalence rates of mental health problems during the perinatal period.

## Abbreviations

AI: artificial intelligence
FIML: full information maximum likelihood
MAR: missing at random
PA: proportion affected
PoI: proportion of interaction
RA: research assistant
REDCap: Research Electronic Data Capture

## Data Availability

The data sets generated during this study are not publicly available as this study is not completed. Data will be available from the corresponding author on reasonable request upon conclusion of the study.
